# Lyon bracing in adolescent females with idiopathic scoliosis: assessment of results according to the SRS committee on bracing and nonoperative management standardization criteria

**DOI:** 10.1186/1748-7161-8-S1-O33

**Published:** 2013-06-03

**Authors:** AG Aulisa, V Guzzanti, P Perisano, G Scudieri, L Bocchino, S Teramo, L Aulisa

**Affiliations:** 1 Orthopaedic Department, Children's Hospital Bambino Gesù, Rome, Italy; 2 Department of Orthopaedics, University Hospital "Agostino Gemelli", Catholic University of the Sacred Heart, Rome, Italy

## Background

The Lyon brace, devised by Stagnara in 1950, is commonly prescribed in many European countries, and is based on the three-point pressure system. The Lyon brace also applies derotational forces to the spine, with the main thoracic plate pushing the hump from the posterior convexity toward the anterior concavity of the scoliotic curve. Lyon bracing is considered to be an effective means for conservative treatment of scoliosis.

## Aim

The purpose of the present study was to evaluate the efficacy of Lyon bracing in the correction of thoracic curves, in agreement with the Scoliosis Research Society (SRS) Committee on Bracing and Nonoperative Management Standardisation Criteria.

## Methods

Sixty-eight adolescent females (mean age 11.8 ± 0.5 years) with a thoracic curve and a pre-treatment Risser score ranging from 0 to 2 were enrolled. All patients were prescribed with full-time Lyon bracing. The minimum duration of follow-up was 24 months (mean: 36.4 ± 27.0 months). Antero-posterior radiographs were used to estimate the curve magnitude (CM) at 5 time points: beginning of treatment (t1), one year after the beginning of treatment (t2), intermediate time between t1 and t4 (t3), end of weaning (t4), 2-year minimum follow-up from t4 (t5). Three situations were distinguished: curve correction, curve stabilization and curve progression.

## Results

CM mean value was 34.1 ± 7.4 SD at t1 and 23.1 ± 10.4 SD at t5. Curve correction was accomplished in 83.8 % of patients, whereas a curve stabilisation was obtained in 16.2 % of patients. None of the patients experienced a curve progression.

## Conclusion

The Lyon brace, due to its biomechanical action on vertebral modelling, is highly effective in correcting thoracic curves.
